# Development of Hourly Indoor PM_2.5_ Concentration Prediction Model: The Role of Outdoor Air, Ventilation, Building Characteristic, and Human Activity

**DOI:** 10.3390/ijerph17165906

**Published:** 2020-08-14

**Authors:** Chien-Cheng Jung, Wan-Yi Lin, Nai-Yun Hsu, Chih-Da Wu, Hao-Ting Chang, Huey-Jen Su

**Affiliations:** 1 Department of Public Health, China Medical University, Taichung 40402, Taiwan; a2468126@gmail.com; 2 Department of Environmental and Occupational Health, College of Medicine, National Cheng Kung University, 138 Sheng-Li Road, Tainan 70403, Taiwan; wanyi821019@gmail.com (W.-Y.L.); boni.hsu@gmail.com (N.-Y.H.); oliverex800630@hotmail.com (H.-T.C.); 3 Department of Geomatics, National Cheng Kung University, Tainan 70403, Taiwan; chidawu@mail.ncku.edu.tw; 4 National Institute of Environmental Health Sciences, National Health Research Institutes, Miaoli 35053, Taiwan

**Keywords:** indoor air, multiple linear regression model, PM_2.5_

## Abstract

Exposure to indoor particulate matter less than 2.5 µm in diameter (PM_2.5_) is a critical health risk factor. Therefore, measuring indoor PM_2.5_ concentrations is important for assessing their health risks and further investigating the sources and influential factors. However, installing monitoring instruments to collect indoor PM_2.5_ data is difficult and expensive. Therefore, several indoor PM_2.5_ concentration prediction models have been developed. However, these prediction models only assess the daily average PM_2.5_ concentrations in cold or temperate regions. The factors that influence PM_2.5_ concentration differ according to climatic conditions. In this study, we developed a prediction model for hourly indoor PM_2.5_ concentrations in Taiwan (tropical and subtropical region) by using a multiple linear regression model and investigated the impact factor. The sample comprised 93 study cases (1979 measurements) and 25 potential predictor variables. Cross-validation was performed to assess performance. The prediction model explained 74% of the variation, and outdoor PM_2.5_ concentrations, the difference between indoor and outdoor CO_2_ levels, building type, building floor level, bed sheet cleaning, bed sheet replacement, and mosquito coil burning were included in the prediction model. Cross-validation explained 75% of variation on average. The results also confirm that the prediction model can be used to estimate indoor PM_2.5_ concentrations across seasons and areas. In summary, we developed a prediction model of hourly indoor PM_2.5_ concentrations and suggested that outdoor PM_2.5_ concentrations, ventilation, building characteristics, and human activities should be considered. Moreover, it is important to consider outdoor air quality while occupants open or close windows or doors for regulating ventilation rate and human activities changing also can reduce indoor PM_2.5_ concentrations.

## 1. Introduction

Particulate matter with an aerodynamic diameter of fewer than 2.5 µm (PM_2.5_) is a critical risk factor for hospital admission for respiratory [1,2] and cardiovascular diseases [1,3]. PM_2.5_ exposure is also associated with diabetes [4,5]. According to statistical data, individuals spend over 80% of their time indoors [6,7], particularly in their homes. Studies have indicated that PM_2.5_ levels in homes are associated with variation in cardiovascular disease indexes [8,9] and those metal elements or polycyclic aromatic hydrocarbons in PM_2.5_ increase carcinogenic risk [10,11,12]. Therefore, measuring indoor PM_2.5_ concentrations to study influential factors, sources, or health effects is crucial.

In countries such as the United States (https://www.epa.gov/outdoor-air-quality-data) and Japan (https://www.airvisual.com/japan), obtaining real-time outdoor PM_2.5_ data from measurement networks is easy. In Taiwan, 78 air quality monitoring stations have been established since 1996 to measure outdoor PM_2.5_ concentrations (https://airtw.epa.gov.tw/ENG/default.aspx), and PM_2.5_ data are available on Taiwan’s Environmental Protection Administration (EPA) website. However, obtaining indoor PM_2.5_ data is difficult because of complications that often arise in the long term after installing sampling instruments in crowded indoor areas, particularly in homes. Moreover, sampling instruments are expensive, and samples containing a few homes may not be representative of exposure situations. Therefore, some studies developed prediction models for indoor PM_2.5_ concentrations. Yuchi and Clark [13,14] used a multiple linear regression (MLR) model to predict indoor home PM_2.5_ levels. Tang and his co-workers used the mass balance method to create a prediction model for indoor PM_2.5_ levels in homes [15]. Elbayoumi used an MLR model to estimate indoor PM_2.5_ levels in schools [16]. However, these studies were conducted in temperate or cold regions, and air conditioning use or the influence of outdoor air on indoor air may differ by climate region. For example, Lai and his co-workers noted that the climate was warmer in south China than in north China and that natural ventilation usage frequency was higher in south China [17]. Studies have demonstrated that indoor air quality differs by ventilation type [18,19,20]. Our previous study observed that outdoor air more critically affects indoor air quality in tropical and subtropical regions than in cold or temperate regions [21], particularly during cold seasons. Outdoor air is a critical source of PM_2.5_ in homes [13,22], and indoor sources, such as cigarettes, cooking, or coal smoke from home heating stoves, are major indoor sources of PM_2.5_ [13,14,15]. In tropical and subtropical regions, buildings are designed to reduce indoor heat and facilitate air conditioning operations for several hours, whereas in temperate regions, buildings are designed to trap heat. Therefore, influencing factors, such as air conditioning usage and building characteristics, differ by region. The indoor PM_2.5_ concentration is also a concern in tropical and sub-tropical regions [23,24,25]. Therefore, developing an indoor PM_2.5_ concentration prediction model to rapidly obtain data is necessary.

Previous studies have estimated the prediction model for daily average PM_2.5_ levels [13,14,15,16]. However, these models do not consider short-term concentration fluctuations. Some studies have estimated hourly indoor aerosol concentrations based on the mechanisms of aerosol [26,27,28]. These models consider aerosol characteristics, but ignore other factors influencing the PM_2.5_ concentration, such as the ventilation condition and cleaning activities. Moreover, the building type or height is a major factor influencing indoor air quality [29,30,31]. Thus, collecting information on human activities and building characteristics and developing a model for predicting hourly average PM_2.5_ concentrations in different climatic regions are necessary to identify factors influencing the PM_2.5_ concentration and particle pollutant epidemiology.

Dampness in Buildings and Health (DBH) is a cross-country project that investigates the association between chemical and biological pollutant exposure and childhood allergies, asthma, and reported symptoms. We are part of DBH’s research team and conducted a study from the period 2008 to 2009 in Tainan, Taiwan [32,33], which is located between tropical and subtropical regions. The study investigated building characteristics, indoor and outdoor air quality, and human activities from 93 households. We used data from the DBH study to (1) create a prediction model for hourly average indoor PM_2.5_ concentrations and (2) analyze the factors influencing indoor PM_2.5_ concentrations. These results can prove beneficial for studying the relationship between PM_2.5_ exposure and health effects and can help the government in formulating policies for reducing indoor PM_2.5_ concentrations.

## 2. Material and Methods

### 2.1. Study Area and Design

**Figure 1:**
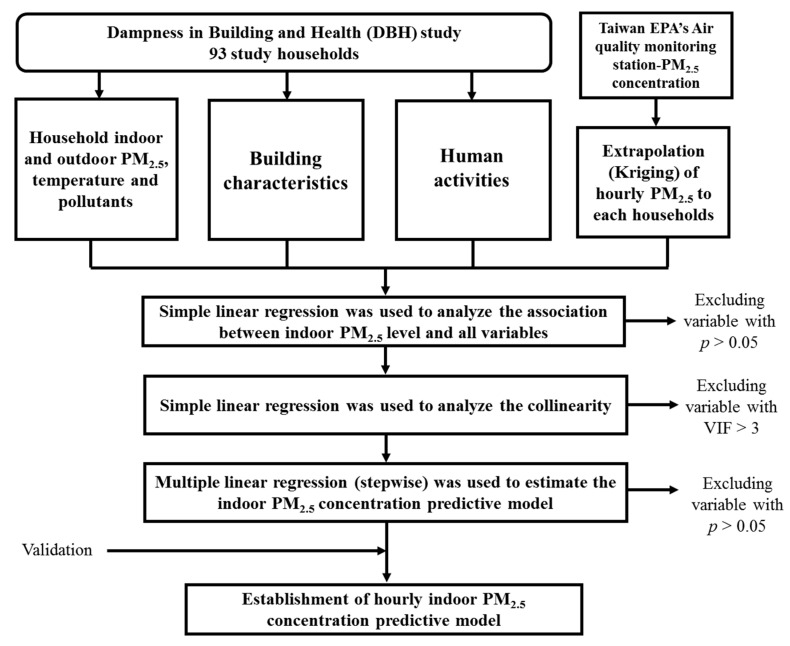
Flowchart of developing the hourly indoor particulate matter (PM)_2.5_ concentration prediction model.

The study framework is presented in Figure 1. The data were used to analyze from the DBH study in Tainan, Taiwan. Tainan (120°38′ E; 120°01′ E; 22°53′ N; 23°24′ N; area, 2199 km^2^) is a city in southwest Taiwan. It is the sixth largest city in Taiwan, with a population of over 1.88 million people. The climate region is split between tropical and subtropical regions. The annual temperature, relative humidity, and rainfall are 24.3 °C, 77.2%, and 1698 mm [34], respectively.

In brief, the sample comprised 93 children (the DBH study had 101 participants but eight were excluded because of missing PM_2.5_ data) aged from 3 to 9 years who completed questionnaires regarding the building characteristics of children’s bedrooms, human indoor activities, furniture materials, and disease history in Tainan, Taiwan, from August 2008 to September 2009. The number of study cases was 21, 39, 13, and 20 in the spring, summer, fall, and winter, respectively. The indoor air quality (including PM_2.5_, CO_2_, and temperature) of the children’s bedrooms was recorded during visits. The samplers also simultaneously collected outdoor air quality, and the hourly outdoor PM_2.5_ concentrations of each household was also modeled using the Kriging model. We used a simple linear model regression to select variables, and MLR was used to estimate a final model. This study also employed cross-validation to assess the prediction model.

### 2.2. Data Collection and Source

#### 2.2.1. Building Characteristics, Human Indoor Activities, and Furniture Materials

During each visit of DBH study, questionnaires were used to survey building characteristics (e.g., building age and type), human indoor activities (e.g., smoking, incense burning, or planting), and furniture materials (e.g., wood or leather) during visits. All sources of potential predictors are shown in Table S1. The data are summarized in Table 1 and Table 2.

#### 2.2.2. Indoor and Outdoor Air Quality

During each visit of DBH study, to prevent the occupants’ daily activities, such as walking, from being obstructed, the indoor PM_2.5_ instruments were located near the wall of children’s bedrooms, and the instruments’ inlet was directed toward the air in their bedrooms. Another PM_2.5_ instrument was simultaneously used to collect data of outdoor PM_2.5_ concentrations on the balconies of children’s bedrooms during the same visits. When outdoor PM_2.5_ instruments were not available on the balconies, the sampler was attached to the windows of children’s bedrooms and their inlets were used to connect the tube for outdoor sampling. PM_2.5_ instruments’ inlets were located 1.0 to 1.2 m above floor level for indoor and outdoor sampling. The concentrations of indoor and outdoor PM_2.5_ were detected using DUST-TRAK aerosol monitors (Model 8520; TSI Corporation, Shoreview, MN, USA). The instruments automatically recorded data at 5-min intervals for 22–24 h. We then downloaded the data from instruments and analyzed the hourly average PM_2.5_ concentration. We recorded 1979 measurements for hourly indoor and outdoor PM_2.5_ concentrations. Before sampling, we compared the two DUST-TRAK aerosol monitors in the same space, and in each measurement, R^2^ was required to exceed 0.995.

DUST-TRAK aerosol monitoring is a type of light-scattering aerosol measuring device. Previous studies have indicated that the data from the DUST-TRAK aerosol monitoring system could be overestimated when the relative humidity is greater than 60% [35,36,37]. The average relative humidity is greater than 75% in Taiwan. Thus, PM_2.5_ samples were simultaneously collected through both personal environmental monitoring with an air flow of 10 L/min on 37-mm Teflon filters and DUST-TRAK aerosol monitoring. In total, indoor and outdoor air quality measurements were collected from 15 and 12 study households, respectively (three outdoor samples had missing data), to calibrate the data from the DUST-TRAK aerosol monitors, respectively. The filters were weighed before and after sampling and stored in a room with controlled temperature at 23 ± 3 °C and relative humidity at 40 ± 5% for at least 24 h. The sampling time for each sample was 24 h. The calibration equations were calculated as Equations (1) and (2) for indoor and outdoor PM_2.5_, respectively, by using a linear regression model.

Y = 0.2118 ∗ DUST-TRAK (Indoor air) + 5.5798; R^2^ = 0.86 (1)

Y = 0.2859 ∗ DUST-TRAK (Outdoor air) + 5.6725; R^2^ = 0.82 (2)

To reduce the difficulty of collecting variables for a prediction model of indoor PM_2.5_ concentration, we collected outdoor PM_2.5_ concentrations near the study households from Taiwan’s EPA and calculated the outdoor PM_2.5_ levels of study cases through the Kriging model [38]. Pearson correlation analysis revealed a significant association (r = 0.91, *p* < 0.0001) between outdoor PM_2.5_ concentrations of the Kriging model and the study households.

To develop a prediction model for predicting indoor PM_2.5_ concentrations, we also used two Q-TRAK air quality monitors (Model 7575; TSI Corporation, Shoreview, MN, USA) to measure indoor and outdoor CO_2_ and temperatures, respectively. The instruments automatically recorded data at 5-min intervals for 22–24 h. We downloaded the data from instruments and analyzed the hourly average concentration. The sampling strategies were consistent with the descriptions provided in this subsection.

### 2.3. MLR Model Procedure

The MLR was used to select variables to establish a prediction model for indoor PM_2.5_ concentrations. The process was as follows:

- Step1: A simple regression analysis was used to analyze the association between indoor PM_2.5_ level and all variables. Variables with *p* > 0.05 were excluded;
- Step 2: A simple linear regression model was used to assess the collinearity between variables. Values with a variance inflation factor >3 were excluded to establish the prediction model;
- Step 3: MLR (stepwise) was used to analyze the association between all variables and indoor PM_2.5_ concentrations. We repeated this process until no more variables could be removed without statistically significant (*p* > 0.05) changes in the regression model.

### 2.4. Prediction Model Performance Evaluation

Prediction model performance was assessed using the coefficient of determination (R^2^) and root mean square error. We used 5-fold cross-validation (80% data for development, 20% for validation) to confirm model reliability. This study also used the developed prediction model to calculate the indoor PM_2.5_ concentrations and further analyzed the corrections between predicted PM_2.5_ concentrations and measured PM_2.5_ concentration to investigate the performance of the final prediction model in different seasons and areas. SAS (v9.4, SAS Institute Inc., Cary, NC, USA) statistical software was used to analyze all data.

## 3. Results

### 3.1. Building Characteristics and Human Activity

Table 1 indicates that townhouses (57%) were the major building type and window and single-split air conditioners were major types of air conditioners (59%). Most buildings were 20–40 years old and had more than three floor levels. Furthermore, 45% of buildings were located near a main road and 84% of households had painted walls. Most of the furniture was made from wood (92%).

As presented in Table 2, 20%, 39%, 48%, and 15% of occupants indulged in planting, smoking, incense burning, and indoor mosquito coil burning. Furthermore, the floors of 65% of children’s rooms were cleaned daily. The furniture in 60% of children’s rooms was cleaned every month. The bed sheets in children’s rooms were cleaned or replaced every month in 58% and 56% households, respectively. Other minor building characteristics and human indoor activities are presented in Table 1 and Table 2.

### 3.2. Distribution of Indoor and Outdoor PM_2.5_ Level

Table 3 lists the distribution of indoor and outdoor PM_2.5_ levels. Average concentrations of indoor and outdoor PM_2.5_ were 19.5 ± 10.6 (DUST-TRAK) and 38.1 ± 20.6 µg/m^3^ (Kriging), respectively. The highest (27.4 ± 12.0 µg/m^3^, DUST-TRAK) and lowest (13.7 ± 6.5 µg/m^3^, DUST-TRAK) levels of indoor PM_2.5_ occurred during the winter and summer, respectively. The highest (53.0 ± 22.3 µg/m^3^, Kriging) and lowest (25.1 ± 11.3 µg/m^3^, Kriging) levels of outdoor PM_2.5_ occurred during the winter and summer, respectively. Seasonal distributions of outdoor PM_2.5_ concentrations were consistent with those of indoor PM_2.5_. The levels of indoor and outdoor temperature and CO_2_ are presented in Table S2. The highest and lowest indoor temperatures were observed in the summer and winter, respectively, similar to the results for outdoor temperature. The highest and lowest levels of CO_2_ were observed during the winter and spring, respectively. However, the seasonal change was not obvious.

### 3.3. MLR Model Results

The results of the MLR model are presented in Table 4. Our model indicated that the outdoor PM_2.5_ concentrations (Kriging), difference in indoor and outdoor CO_2_ levels, building types, building floor levels, the frequencies of bed sheet cleaning and replacing, and mosquito burning behavior were associated with the indoor PM_2.5_ concentrations. The overall R^2^ was 74%. The equation of indoor PM_2.5_ concentration prediction model is as follows

Indoor PM_2.5_ (µg/m^3^) = (Outdoor PM_2.5_ (µg/m^3^)) × 0.422 − 0.003 × (difference of indoor and outdoor CO_2_ (ppm)) + 0.565 × (building type) − 1.292 × (building floor level) + 1.310 × (frequency of clean ned sheet) + 1.166 × (frequency of replace bed sheet) + 2.318 × (mosquito coil burning) (3)

### 3.4. Validation Result

**Figure 2:**
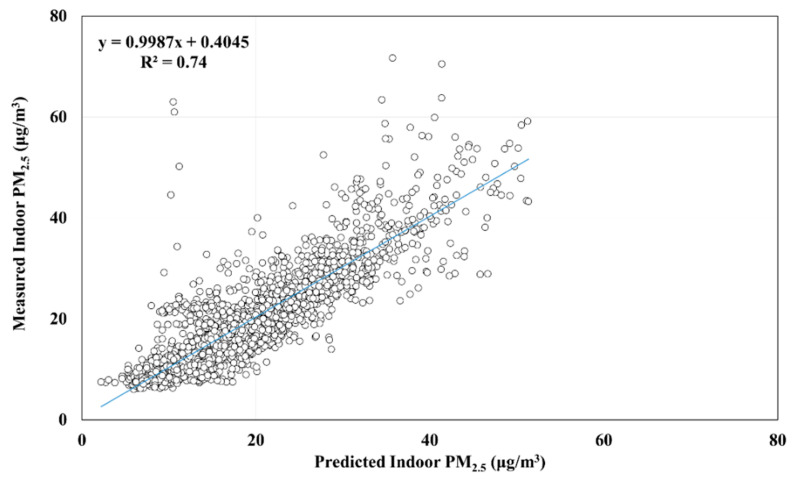
Relationship between predicted and measured hourly average indoor PM_2.5_ concentration according to the multiple linear regression model.

**Figure 3:**
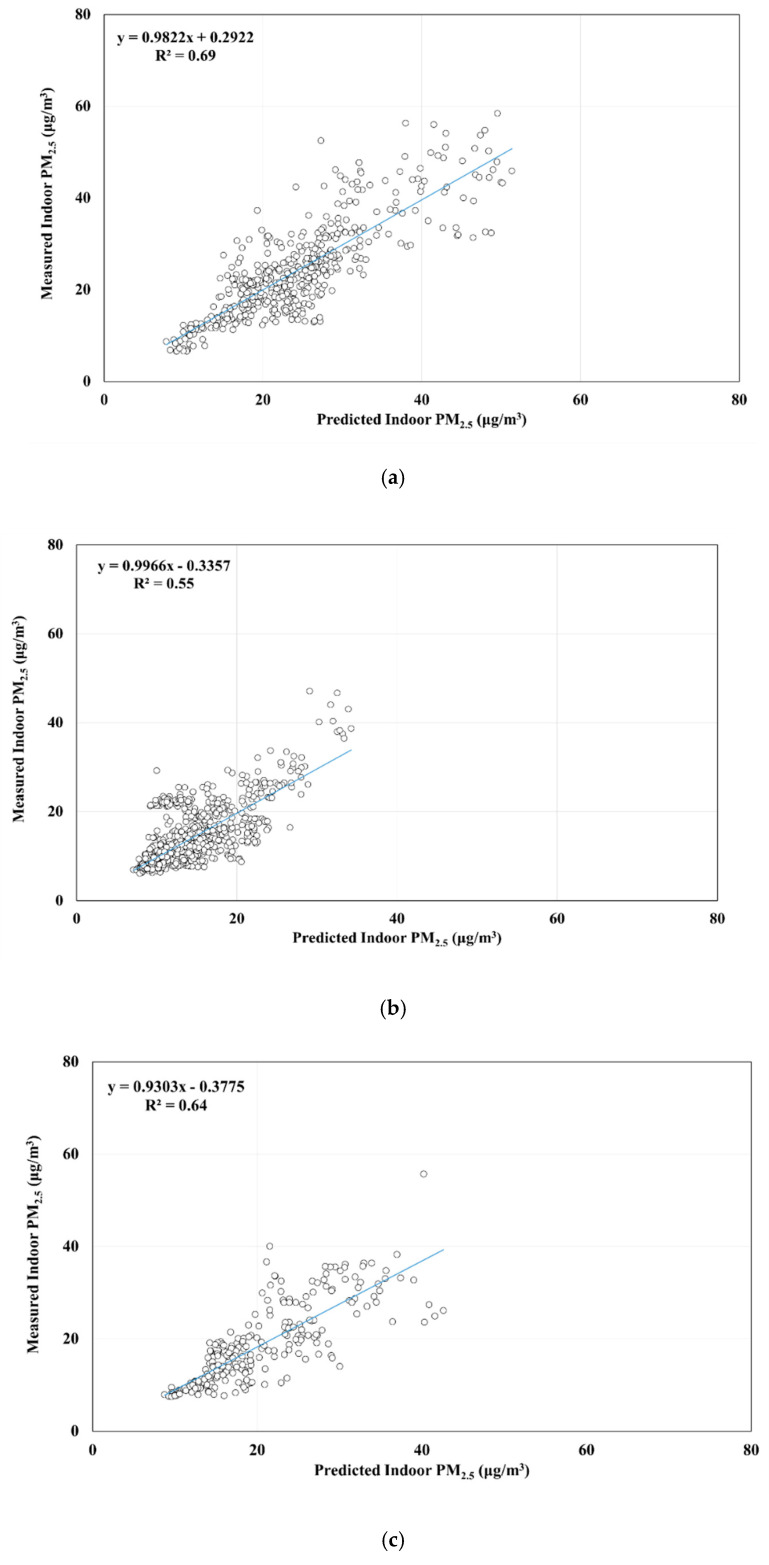

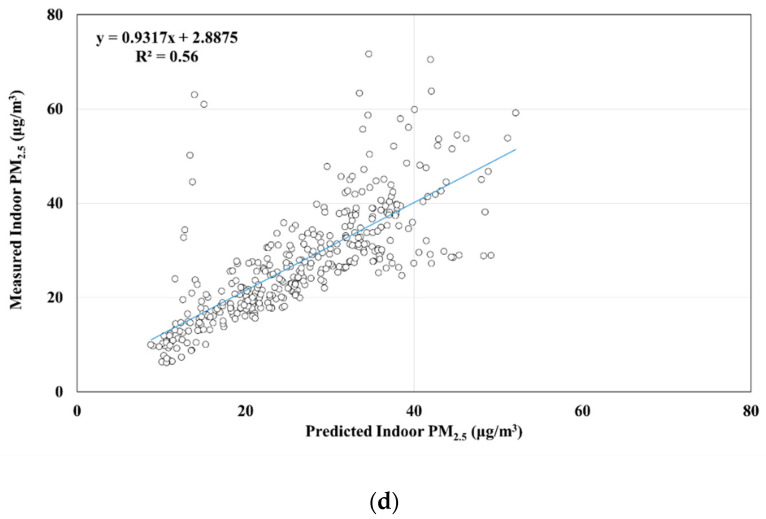
Relationship between predicted and measured hourly average indoor PM_2.5_ concentration across seasons: (**a**) spring, (**b**) summer, (**c**) fall, and (**d**) winter.

**Figure 4:**
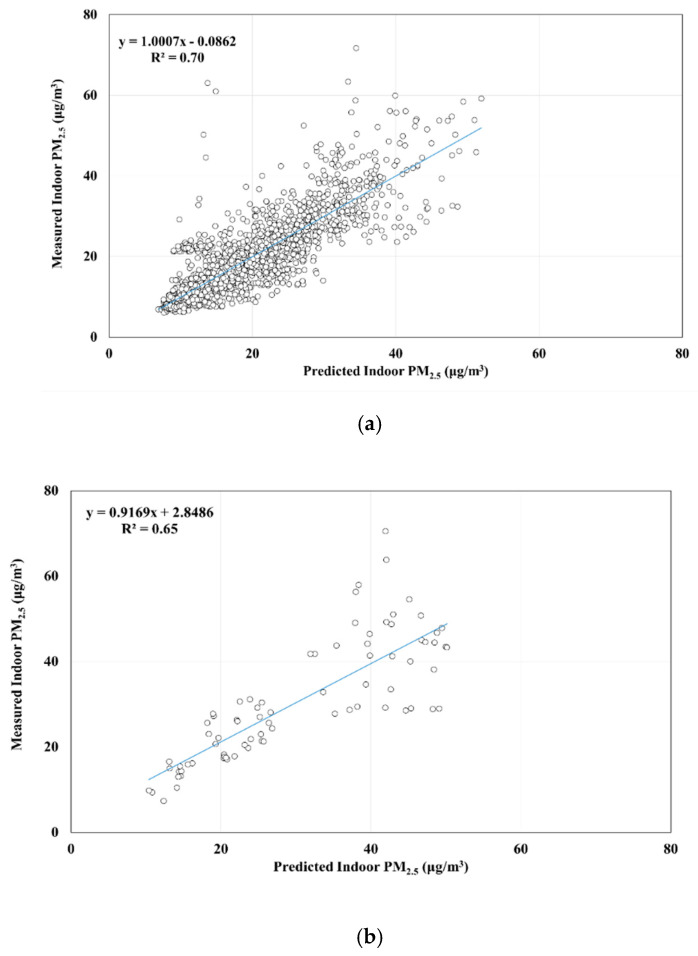
Relationship between predicted and measured hourly average indoor PM_2.5_ concentration across areas: (**a**) urban and (**b**) nonurban areas.

Table 5 presents the results of cross-validation for the MLR model for hourly average indoor PM_2.5_ concentrations. As presented in Table 5, we used 20% of the data to validate the predicted model, which indicated that the adjusted R^2^ had a range of 72–78%, with an average of 75%. Figure 2 displays the results of a Pearson correlation analysis, which revealed a significant relationship between predicted and measured indoor PM_2.5_ concentrations (R^2^ = 0.74, *p* < 0.05). We also used Equation (3) to calculate the indoor PM_2.5_ concentrations and further analyzed the associations between predicted and measured values by season and area (Figure 3 and Figure 4). The results of a Pearson correlation analysis revealed a satisfactory association between predicted and measured indoor PM_2.5_ concentrations (*p* < 0.05) in different seasons or areas, thus indicating that the prediction model sufficiently estimated hourly average indoor PM_2.5_ concentrations.

### 3.5. Discussion

We created a prediction model for hourly average indoor PM_2.5_ concentrations by using MLR model, the results of which suggested that outdoor PM_2.5_, ventilation, building characteristics, and human activities were key predictors. This prediction model also sufficiently estimated hourly average PM_2.5_ concentrations in different seasons and areas. Based on the modeling results, we suggest that controlling outdoor PM_2.5_ emission and certain human activities are beneficial for reducing indoor PM_2.5_ concentrations. Occupants should also consider the outdoor air quality when they open or close windows or doors to regulate ventilation. This prediction model can be used to assess variations in indoor PM_2.5_ concentrations across seasons and areas for future epidemiological research and make the control policy.

The average age of buildings in Taiwan is 29.6 years, and 54% of buildings are aged 20–40 years [39]. In this study, the average building age was 27.0 years, and 62% were aged between 20–40 years. Moreover, 50%, 18%, and 32% of people in Taiwan live in townhouses, single-family homes, and apartments, respectively, according to statistical data [39]. The households in our study were similarly distributed across building types (Table 1). These data reflected that building type and age distributions from the studied households matched the situation in Taiwan.

In this study, both indoor and outdoor PM_2.5_ concentrations were the highest in the winter (*p* < 0.05, based on analysis of variance), and the next highest of concentration in spring. This reveals that indoor and outdoor PM_2.5_ pollution is more serious in the winter and spring than that in the summer and fall in Taiwan. Studies have indicated that dust storms, biomass burning, and long transportation from China are notable contributors to outdoor PM_2.5_ in the winter or spring [40,41,42]. Kuo and Shen [41] found that indoor and outdoor PM_2.5_ concentrations were high during dust-storm periods. Moreover, our prediction model indicated that outdoor PM_2.5_ concentrations was a critical factor affecting indoor PM_2.5_ concentrations. This explains why high indoor and outdoor PM_2.5_ concentrations were observed in the spring and winter, and similar seasonal patterns.

Table 4 indicates that outdoor PM_2.5_ concentrations is a critical parameter in the indoor PM_2.5_ concentration prediction model. In Taiwan, opening windows is a universal behavior, and indoor PM_2.5_ concentration is associated with outdoor PM_2.5_ concentration in residential areas [41,43]. Previous studies have also indicated that outdoor PM_2.5_ is a major variable in indoor PM_2.5_ prediction models [22,44]. Therefore, outdoor PM_2.5_ concentration plays an important role in predicting indoor PM_2.5_ concentrations.

Some studies directly measured outdoor PM_2.5_ concentrations to predict indoor PM_2.5_ concentrations [13,16]; however, installing sampling instruments to measure outdoor PM_2.5_ concentration in studied households for the prediction of indoor PM_2.5_ concentration is difficult. Studies have directly collected outdoor PM_2.5_ data from monitoring stations [14,15]; however, given the distance between monitoring stations and studied households, the data from monitoring stations have a large error and do not accurately reflect the pollution levels near the study households. In this study, we used the Kriging model to estimate the outdoor PM_2.5_ concentrations surrounding studied households. Chiang used a Kriging model to estimate outdoor PM_2.5_ concentration in Taiwan [45]. The data from Taiwan’s EPA air quality–monitoring stations were critical for simulating outdoor PM_2.5_ concentrations by using the Kriging model, and these data were easily collected online. Moreover, a satisfactory correlation was observed between the outdoor PM_2.5_ concentrations surrounding studied households determined using the Kriging model and measurements in this study (r = 0.91, *p* < 0.0001). Therefore, we can directly collect outdoor PM_2.5_ concentrations from Taiwan’s EPA air quality monitoring stations and estimated the outdoor PM_2.5_ concentrations of studied households by using the Kriging model. These data from the Kriging model can be inputted in the prediction model for calculating the indoor PM_2.5_ concentrations.

Our data indicate that indoor PM_2.5_ concentration is negatively associated with the differences of indoor and outdoor CO_2_ levels in Table 4, and building type was also a factor influencing the indoor PM_2.5_ concentrations. In Langer and Bekö’s study [30], they found that ventilation rate was positively associated with the level of indoor NO_2_ level. Outdoor air was an important contributor to NO_2_ in indoor air, due to high ventilation rate increasing the contribution of outdoor NO_2_ to indoor air. Hänninen and co-workers indicated that the ventilation rate was positively associated with the PM_2.5_ infiltration rate [46]. Our model also indicated that outdoor PM_2.5_ was an important factor influencing indoor PM_2.5_ concentrations. In this study, the average differences of indoor and outdoor CO_2_ concentrations were 359, 284, and 256 ppm in buildings with townhouse, single-family, and apartment, respectively. High ventilation rate may increase the contribution of outdoor PM_2.5_ to indoor air. We determined that apartments contribute more to the indoor PM_2.5_ concentration than single-family houses and townhouses do. We speculated that the small space of the apartments has a large cumulative effect on the PM_2.5_ concentration. Our data indicated that the indoor PM_2.5_ concentrations was higher on the first floors than on other floors. We speculated that emissions from vehicular emission was a critical factor influencing the indoor PM_2.5_ concentration [29]. Therefore, the first-floor level had a higher risk of exposure to higher PM_2.5_. Taken together, building characteristics were important influence factors and occupants should consider the outdoor air quality when they regulate the ventilation situation.

Table 4 presents frequencies of bed sheet cleaning and replacing are also predictors of indoor PM_2.5_ concentrations. One study investigated the variations in particulate matter concentrations for different human activities indoors and found that a folded blanket also contributed particulate matter due to resuspension [28]. We speculated that particle concentration was higher on the bed sheet when the frequency of bed sheet cleaning or replacing was lower, and occupants’ use or a folded bed sheet increase the particle concentrations due to resuspension. Previous studies have also demonstrated that mosquito coil burning was a contributor to particulate matter in the air [47,48]. Mosquito bite is universal in tropical and subtropical regions, and a mosquito coil is often used to prevent mosquito bite. Therefore, we cannot ignore the effect of mosquito coil burning on indoor air quality.

Studies have indicated that other indoor human activities, such as smoking or incense stick burning, are major factors affecting the PM_2.5_ concentration [47,48,49]. Our data indicated that indoor PM_2.5_ concentrations are not associated with smoking and incense stick burning. We speculate that the burning time of cigarettes and incense sticks was shorter and the effects of cigarette and incense stick burning was weaker on indoor PM_2.5_ concentrations. Moreover, this study lacked detailed records of the occupants’ behaviors and the exact time of event. Applying feasible technology to collect occupant activity frequency and time is necessary for completely analyzing the effects of occupant activities on the hourly PM_2.5_ concentration in the future.

Studies have completed cross-validation for prediction models [13,15,50]. In this study, we also conducted cross-validation, which revealed that R^2^ > 75%. Figure 3 and Figure 4, respectively, illustrate significant correlations between predicted and measured indoor PM_2.5_ concentrations in different seasons and areas. These correlations were significant, and the R^2^ values ranged from moderately correlated to highly correlated, which indicates that the prediction model in this study can be used to estimate indoor PM_2.5_ concentrations in across seasons and areas in Taiwan. However, Figure 2, Figure 3 and Figure 4 show some measurements have larger relative errors, especially in winter. In Sun’ study [51], they found that window opening/closing behavior significantly affected the error on the prediction model of indoor PM_2.5_ concentration. Our study did not investigate the time point and frequency of window opening/closing behaviors. Thus, the time point or frequency of window opening/closing behaviors investigation was necessary in the near future for reducing the relative error.

Some studies developed indoor aerosol modeling according to the complicated mechanisms (such as coagulation or secondary formation) [26,27,28]. However, these models could ignore the building characteristics or other human activities. Some studies estimate prediction models for indoor PM_2.5_ concentration in households [13,14,15], but these studies were conducted in temperate or cold regions, such as in Canada, Mongolia, and the United States. The predictors in our study differed from those in previous studies. For example, in the studies by Clark and Yuchi, outdoor PM_2.5_ concentrations was not a crucial predictor. We speculate that outdoor air does not critically influence indoor air in different temperature regions because of doors and windows closing, particularly in the winter. In Taiwan, the frequency of window-opening behavior is high due to its warm climate [21]; thus, outdoor air considerably influences indoor air in Taiwan. Moreover, Clark’s study indicated that forced air heating is a key variable in prediction models of indoor PM_2.5_ concentrations. We did not find that heater use was associated with indoor PM_2.5_ concentration, possibly because only 8% of occupants had heaters; moreover, the heaters were only operated 0.3 h/day during the winter in our previous study [21]. This indicates that heater use is not a critical variable in tropical and subtropical regions. Altogether, human activity may differ across climate regions and influence indoor air quality with different predictors.

## 4. Conclusions

This study used the data on household indoor and outdoor pollutants, building characteristics, and human indoor activity from the DBH study in Taiwan to develop a prediction model for hourly indoor PM_2.5_ concentrations and further investigate the impact factor. Our results revealed that outdoor PM_2.5_ concentrations, difference between indoor and outdoor CO_2_ levels, building type, building floor level, bed sheet cleaning, bed sheet replacing, and mosquito coil burning were key variables, and the predictor was not different on different climate regions. The prediction model of indoor PM_2.5_ concentrations can explain 74% of the variation and sufficiently predict indoor PM_2.5_ concentrations across seasons (55 to 69%) and areas (65 to 70%). PM_2.5_ concentrations from Taiwan’s EPA air quality monitoring stations can be used to estimate the outdoor PM_2.5_ concentrations of studied households by using the Kriging model and thereby reduce the time and cost of installing PM_2.5_ monitoring instruments.

This study developed a prediction model for predicting indoor hourly PM_2.5_ concentrations in tropical and subtropical regions, and found that the major predictors were different according to different climate regions. Controlling outdoor PM_2.5_ pollution emission and changing certain human activities can reduce PM_2.5_ exposure. Occupants should assess the outdoor air quality when they open or close doors or windows for ventilation rate regulation. We assert that our prediction model can estimate indoor PM_2.5_ concentrations across seasons and areas in Taiwan and can be used for future epidemiological research on the relationship between indoor PM_2.5_ exposure and health effects and obtaining rapid data for pollution control.

## Figures and Tables

**Table 1:** Summary of building characteristics and materials of 93 studied households.

Variables	Description	Results (*n*, %)
Building characteristics		
Building type	Townhouse	53 (57%)
	Single-family	14 (15%)
	Apartment	26 (28%)
Building age	<10 years	3 (3%)
	10–20 years	26 (29%)
	20–40 years	56 (62%)
	>40 years	6 (6%)
Building floor level	1	7 (7%)
	2	35 (38%)
	>3	51 (55%)
Near main road (<8 m) ^&^	Yes	42 (45%)
Air conditioner type	Central air conditioner	29 (31%)
	Window or single-split air conditioner	55 (59%)
	None	9 (10%)
Heater	Yes	7 (8%)
Wall material		
Wall paint	Yes	78 (84%)
Furniture material		
Wood	Yes	86 (92%)
Cloth	Yes	6 (6%)
Leather	Yes	2 (2%)
Imitation leather	Yes	3 (3%)
Plastic	Yes	35 (38%)
Iron or glass	Yes	26 (28%)

^&^ The distance between study households and main road is less than 8 m. According to Taiwan’s Design Directions of Urban Roads and Accessory Works, the main road is the road that connects towns.

**Table 2:** Summary of human activities of 93 studied households.

Variables	Description	Results (*n*, %)
Planting *	Yes	19 (20%)
Smoking *	Yes	36 (39%)
Incense stick burning *	Yes	45 (48%)
Mosquito coil burning *	Yes	14 (15%)
Floor cleaning (frequency) ^#^	Every day	60 (65%)
	1 time every 2 weeks	16 (17%)
	1 time per month	17 (18%)
Furniture cleaning (frequency) ^#^	1 time per week	37 (40%)
	1 time per month	56 (60%)
Clean bed sheet (frequency) ^#^	1 time every 2 weeks	39 (42%)
	1 time per month	54 (58%)
Replace bed sheet (frequency^) #^	1 time every 2 weeks	41 (44%)
	1 time per month	52 (56%)

* Human activity during the periods of measurement. ^#^ The frequency of human activities of study households in universal situations.

**Table 3:** Summary of indoor and outdoor PM_2.5_ (µg/m^3^) concentrations (mean ± SD.)

Pollutants	Overall (*N* = 1979)	Spring (*N* = 477)	Summer (*N* = 869)	Fall (*N* = 256)	Winter (*N* = 377)
Indoor PM_2.5_-DUST-TRAK	19.5 ± 10.6	24.2 ± 10.3	13.7 ± 6.5	18.5 ± 8.6	27.4 ± 12.0
Outdoor PM_2.5_-DUST-TRAK	29.5 ± 20.3	38.9 ± 20.4	17.3 ± 9.4	30.2 ± 17.4	45.0 ± 23.2
Outdoor PM_2.5_-Kriging	38.1 ± 20.6	49.0 ± 19.3	25.1 ± 11.3	40.1 ± 17.0	53.0 ± 22.3

**SD.:** Standard deviation.

**Table 4:** Multiple linear regression models for hourly average indoor PM_2.5_ concentrations.

Predictor	Coefficients	Coefficients (95% CI)	*p*-Value	Adjust R^2^ (%)	RMSE
Air pollutants					
Outdoor PM_2.5_ concentration	0.422	0.410 to 0.434	<0.0001		
Difference of indoor and outdoor CO_2_	−0.003	−0.003 to −0.002	<0.0001		
Building characteristics					
Building type (townhouse = 0, single-family = 1, apartment = 2)	0.565	0.145 to 0.985	<0.05		
Building floor level (first floor level = 0, second floor level = 1, more than three floor level = 2)	−1.292	−1.729 to −0.856	<0.0001		
Human activities					
Clean bed sheet (one time every weeks = 0, one time per month = 1)	1.310	0.727 to 1.893	<0.0001		
Replace bed sheet (one time every weeks = 0, one time per month = 1)	1.166	0.864 to 1.467	<0.0001		
Mosquito coil burning (no = 0, yes = 1)	2.318	1.584 to 3.052	<0.0001		
Overall			<0.0001	74	5.41

RMSE: Root mean square error.

**Table 5:** Cross-validation results for hourly average indoor PM_2.5_ concentration.

Validation	*N*	R^2^ (%)	Adjust R^2^ (%)	RMSE
Validation I	395	74	74	5.70
Validation II	395	77	77	4.74
Validation III	395	77	76	5.03
Validation IV	395	78	78	4.87
Validation V	395	72	72	5.25
Average		76	75	

RMSE: Root mean square error.

## Supplementary Materials

The following are available online at https://www.mdpi.com/1660-4601/17/16/5906/s1, Table S1: Summary of predictors considered as candidates for hourly indoor PM_2.5_ concentration model developing, Table S2: Summary of indoor and outdoor temperature and CO_2_.
