# Supplementary material for: Development of Hourly Indoor PM2.5 Concentration Prediction Model: The Role of Outdoor Air, Ventilation, Building Characteristic, and Human Activity
Source: Int J Environ Res Public Health. 2020 Aug 14;17(16):5906. doi: 10.3390/ijerph17165906 (PMC7460507; doi:10.3390/ijerph17165906)
Supplement: Supplementary file 1 [file ijerph-17-05906-s001.pdf]

Supplemental material

Table S1 Summary of predictors considered as candidates for hourly indoor $PM_{2.5}$ concentration model developing.

Type	Variable	Number or category	Data source	Year
Indoor parameter	Building characteristic (building age, type, floor level, and traffic flow near study case)	3	Questionnaire	2008-2009
	Electric equipment usage (air conditioner and heater)	2	Questionnaire	2008-2009
	Human indoor activity (planting, smoking, incense burning, mosquito coil burning, frequency of floor and furniture, bed sheet clean, and bed sheet replace)	8	Questionnaire	2008-2009
	Wall/furniture material (paint, wood, cloth, leather, imitation leather, plastic, and iron or glass)	7	Questionnaire	2008-2009
	Meteorological data (temperature)	1	Measurement (Q-TRAK)	2008-2009
	Air pollutants (CO_2)	1	Measurement (Q-TRAK)	2008-2009

Table S1 Summary of predictors considered as candidates for hourly indoor $PM_{2.5}$ concentration model developing (continuous).

Type	Variable	Number or category	Data source	Year
Outdoor parameter	Meteorological data (temperature)	1	Measurement (Q-TRAK)	2008-2009
	Air pollutants ($PM_{2.5}$-Kriging and CO_2)	2	Measurement (DUST-TRAK and Q-TRAK: $PM_{2.5}$ and CO_2,)<br>Taiwan's EPA Air quality monitoring station ($PM_{2.5}$-Kriging, NO_2, and SO_2)	2008-2009

Table S2 Summary of indoor and outdoor temperature and CO_2 (mean ± SD.).

	Overall (N = 1979)	Spring (N = 477)	Summer (N = 869)	Fall (N = 256)	Winter (N = 377)
**Indoor Temperature (℃)**	27.9 ± 3.7	27.2 ± 2.4	30.0 ± 2.0	29.9 ± 1.8	22.9 ± 3.5
**Outdoor Temperature (℃)**	27.9 ± 5.8	26.6 ± 4.9	31.1 ± 3.3	30.6 ± 3.0	20.6 ± 5.9
**Indoor CO_2 (ppm)**	722 ± 528	634 ± 405	713 ± 543	648 ± 389	904 ± 653
**Outdoor CO_2 (ppm)**	435 ± 109	446 ± 44	402 ± 136	389 ± 27	530 ± 62
**Difference of indoor and outdoor CO_2 levels**	287 ± 530	188 ± 402	311 ± 564	259 ± 386	374 ± 644

SD.: Standard deviation.
